# 72例肺腺鳞癌临床病理特点和预后因素分析

**DOI:** 10.3779/j.issn.1009-3419.2016.10.03

**Published:** 2016-10-20

**Authors:** 熙 吴, 峻岭 李, 舒兰 陈, 雷 于, 渤彦 杨

**Affiliations:** 1 100021 北京，国家癌症中心/中国医学科学院北京协和医学院肿瘤医院综合科 Medical Record Department, National Cancer Center/Cancer Hospital Chinese Academy of Medical Sciences and Peking Union Medical College, Beijing 100021, China; 2 100021 北京，国家癌症中心/中国医学科学院北京协和医学院肿瘤医院北京内科 Internal Department, National Cancer Center/Cancer Hospital Chinese Academy of Medical Sciences and Peking Union Medical College, Beijing 100021, China; 3 100021 北京，国家癌症中心/中国医学科学院北京协和医学院肿瘤医院病案室 General Department, National Cancer Center/Cancer Hospital Chinese Academy of Medical Sciences and Peking Union Medical College, Beijing 100021, China

**Keywords:** 肺肿瘤, 腺鳞癌, 临床病理, 预后, Lung neoplasms, Adenosquamous carcinoma, Clinicopathology, Prognosis

## Abstract

**背景与目的:**

肺腺鳞癌是肺癌中的一种少见类型，混合有腺癌和鳞癌两种恶性组织成分，侵袭性高、预后差。本研究旨在探讨腺鳞癌的临床病理特点和预后影响因素。

**方法:**

对72例肺腺鳞癌患者的临床病理资料进行回顾性分析，探讨影响患者预后的因素。

**结果:**

全组患者中位生存期位34.7个月，5年生存率为14.9%，肿瘤长径、转移（metastasis, M）分期、肿瘤-淋巴结-转移（tumor-node-metastasis, TNM）病理分期、基因突变、手术对患者预后的影响有统计学意义。

**结论:**

肺腺鳞癌恶性程度高、预后差，应采取手术为主的综合治疗，小分子酪氨酸激酶抑制剂治疗有助于延长患者生存期。

## Abstract (en)

**Background and objective:**

Adenosquamous carcinoma (ASC) is a rare subtype of lung cancer, it is mixed glandular and squamous cell carcinoma with a more aggressive behavior and poor prognosis than the other histologic subtypes. The aim of the study was to explore the clinicopathological characteristics and prognostic factors of ASC.

**Methods:**

A total of 72 patients were enrolled. We investigated clinicalpathological features and prognostic factors retrospectively.

**Results:**

The overall 72 ASC patients' median age was 34.7 months, 5-year survival rate was 14.9%. The influence of tumor size, M stage, and N stage, gene mutation and surgery on the prognosis of patients show statistical significance.

**Conclusion:**

ASC is characterized by both histologic aggressiveness and adverse prognosis. We suggest the comprehensive therapy based on surgery, and given small molecule tyrosine kinase inhibitors (TKIs) treatment may prolong patients' overall survival.

肺腺鳞癌（adenosquamous carcinoma lung cancer, ASC）是非小细胞肺癌的一种特殊类型，定义为肿瘤组织中同时含有腺癌和鳞癌两种恶性组织成分，其中任一成分至少占全部肿瘤的10%^[1]^。ASC与其他亚型的非小细胞肺癌具有相同临床表现，但确诊时常已处于疾病中晚期，更易侵犯血管和胸膜，相对于单一肿瘤组织类型的肺腺癌或鳞癌预后更差^[2, 3]^。本研究回顾性分析中国医学科学院北京协和医学院肿瘤医院2011年1月-2015年12月收治经病理确诊的72例ASC患者临床病理资料和随访结果，探讨影响其生存的预后相关因素，旨在为提高治疗水平和评估预后提供依据。

## 1 资料与方法

### 1.1 一般资料

中国医学科学院北京协和医学院肿瘤医院2011年1月-2015年12月间收治经病理证实的ASC 72例。其中男性44例，女性28例，男女比例为1.57:1，中位年龄60岁（32岁-85岁）。40例吸烟（55.6%），均为重度吸烟者（吸烟指数≥400支/年），吸烟史最短10年，最长50年，平均烟龄31年，不吸烟者32例（44.4%）。13例（18.1%）有肿瘤家族史。无症状体检发现者21例（29.2%），咳嗽40例（55.6%），痰中带血24例（33.3%），胸痛8例（11.1%），胸闷气短9例（12.5%），发热2例（2.8%），声音嘶哑1例（1.4%），发现左额头包块1例（1.4%），发现左颈部结节1例（1.4%）。影像学检查显示周围型肺癌56例（77.8%），中央型肺癌16例（22.2%）。肿瘤位于左肺上叶12例（16.7%），下叶18例（25%）；右肺上叶21例（29.1%），中叶3例（4.2%），下叶16例（22.2%）；侵犯2叶肺者2例（2.8%）。全组患者自然病程为平均2.1个月（0.2个月-10个月）（表 1）。

**1:** 72例原发肺腺鳞癌患者的临床特征 Clinical feathers in 72 cases of patients with ASC

Characteristics	*n*	Percentage (%)
Gender		
Male	44	61.1
Female	28	38.9
Age (yr)		
Mean	60	
Range	32-85	
Smoking history		
Former	40	55.6
Never	32	44.4
Family history of cancer		
Yes	13	18.1
No	59	81.9
Symptom		
Physical examination	21	29.2
Cough	40	55.6
Bloody sputum	24	33.3
Chest pain	8	11.1
Shortness of breath	9	12.5
Fever	2	2.8
Hoarseness	1	1.4
Left frontal lump	1	1.4
Left neck lump	1	1.4
CT features		
Peripheral	56	77.8
Central	16	22.2
Lober location		
Left upper	12	16.7
Left lower	18	25
Right upper	21	29.1
Right middle	3	4.2
Right lower	16	22.2
2 lobes of lung	2	2.8
ASC: adenosquamous carcinoma lung cancer; CT: computed tomography.		

### 1.2 术前诊断和术后病理

术前共54例（75%）行纤维支气管镜刷片检查、病理活检、细针穿刺活检及痰脱落细胞学检查。其中术前确诊腺鳞癌6例；术前诊断为鳞癌16例、腺癌15例、非小细胞癌6例、低分化癌1例，另有10例术前细胞学病理检查为阴性。肿瘤长径最小值1.5 cm，最大值7.9 cm，中位值为3.35 cm。61例行肺癌手术切除肿瘤原发灶，其中在我院进行手术的58例患者术后病理显示：51例侵犯脏层胸膜，7例未累及脏层胸膜。T1、T2、T3、T4期分别为5例（8.6%）、41例（70.7%）、10例（17.2%）、2例（3.5%）；N0、N1、N2、N3期分别为22例（37.9%）、10例（17.2%）、24例（41.4%）、2例（3.5%）。全组患者Ⅰ期17例（23.6%），Ⅱ期13例（18.1%）、Ⅲ期31例（43%）、Ⅳ期11例（15.3%）。33例行基因检测（表 2）：10例表皮生长因子受体（epidermal growth factor receptor, *EGFR*）19突变，1例EGFR 20伴EGFR 21突变，6例EGFR 21突变，2例鼠类肉瘤病毒癌基因（kirsten rat sarcoma viral oncogene homolog, KRAS）12密码子突变，2例间变性淋巴瘤激酶（anaplastic lymphoma kinase, *ALK*）突变，1例人类表皮生长因子受体2（human epidermal growth factor receptor-2, *HER2*）基因20外显子突变，11例无基因突变。

**2:** 72例肺腺鳞癌患者的术前诊断和术后病理 Preoperative diagnosis and postoperative pathology in 72 cases of patients with ASC

Pathological characteristics	*n*	Percentage (%)
Preoperative diagnosis		
SC	16	29.6 (16/54)
AC	15	27.8 (15/54)
NSCLC	6	11.1 (6/54)
Poorly differentiated carcinoma	1	1.9 (1/54)
No cancer cell	10	18.5 (10/54)
ASC	6	11.1 (6/54)
Visceral pleural invasion		
Yes	51	87.9 (51/58)
No	7	12.1 (7/58)
Tumor		
T1	5	8.6 (5/58)
T2	41	70.7 (41/58)
T3	10	17.2 (10/58)
T4	2	3.5 (2/58)
Nodes		
N0	22	37.9 (22/58)
N1	10	17.2 (10/58)
N2	24	41.4 (24/58)
N3	2	3.5 (2/58)
Metastasis		
M0	61	84.7 (61/72)
M1	11	15.3 (11/72)
TNM stage		
Stage Ⅰ	17	23.6 (17/72)
Stage Ⅰa	3	4.2 (3/72)
Stage Ⅰb	14	19.4 (14/72)
Stage Ⅱ	13	18.1 (13/72)
Stage Ⅱa	10	13.9 (10/72)
Stage Ⅱb	3	4.2 (3/72)
Stage Ⅲ	31	43.1 (31/72)
Stage Ⅲa	27	37.5 (27/72)
Stage Ⅲb	4	5.6 (4/72)
Stage Ⅳ	11	15.3 (11/72)
Gene test (mutations)	33	
*EGFR* 19	10	30.3 (10/33)
*EGFR* 20 and *EGFR* 21	1	3.0 (1/33)
*EGFR* 21	6	18.2 (6/33)
*KRAS* 12	2	6.1 (2/33)
*ALK*	2	6.1 (2/33)
*HER*2	1	3.0 (1/33)
WT	11	33.3 (11/33)
SC: squamous cell carcinoma; AC: adenocarcinoma; NSCLC: non-small cell lung cancer; WT: wild type.		

### 1.3 治疗方法

非手术治疗11例，61例行肺癌切除术。手术方式：单个肺叶切除48例（78.7%），其中3例行肺叶楔形切除，2例行袖状切除术；双叶切除8例（13.1%）；1例行右肺中下叶及右肺上叶楔形切除术（1.6%）；左全肺切除4例（6.6%）。共14例肺癌术后患者辅助放疗，未手术者4例接受姑息放疗。2例行瘤床区同步放化疗，9例接受胸部放疗，7例因脑转移行脑放疗，2例接受骨转移灶放疗，2例因左肾上腺转移放疗。51例（70.8%）接受辅助化疗，21例（29.2%）单纯手术未化疗。化疗方案：紫杉醇+顺铂/卡铂；吉西他滨+顺铂；培美曲塞+顺铂/卡铂/奈达铂；多西他赛+顺铂/卡铂/贝伐单抗；伊立替康+异环磷酰胺；白蛋白紫杉醇；长春瑞滨+顺铂+恩度；西妥昔单抗+多西他赛+顺铂。

全组患者中有15例（20.8%）因复发转移口服小分子酪氨酸激酶抑制剂（tyrosine kinase inhibitors, TKIs）治疗，每日口服吉非替尼150 mg，厄洛替尼250 mg，埃克替尼375 mg或克唑替尼500 mg。10例疗效较好，疾病无进展时间（progression-free survival, PFS）超过6个月。其中2例ALK阳性患者口服克唑替尼疗效显著，1例PFS 14个月至今生存，1例PFS 30个月后出现脑转移。其余5例无效，2个月内疾病进展死亡。

### 1.4 统计学方法

采用SPSS 22.0软件进行统计分析。*Kaplan*-*Meier*法计算中位生存期、生存率及绘制生存曲线，生存率组间比较采用*Log*-*rank*检验。*P*＜0.05为差异有统计学意义。

## 2 结果

随访至2016年7月或死亡，7例失访，随访率90.3%。随访时间1个月-61个月，生存病例中位随访时间28个月。截止至最后一次随访，56.9%（37/65）的患者死亡。

### 2.1 治疗效果

2例患者围手术期因呼吸衰竭死亡。全组患者中位生存期34.7个月，1年、3年、5年生存率分别为78.5%、47.1%、14.9%（图 1A）。61例手术患者中位生存期37个月，5年生存率17.8%。

**1:**
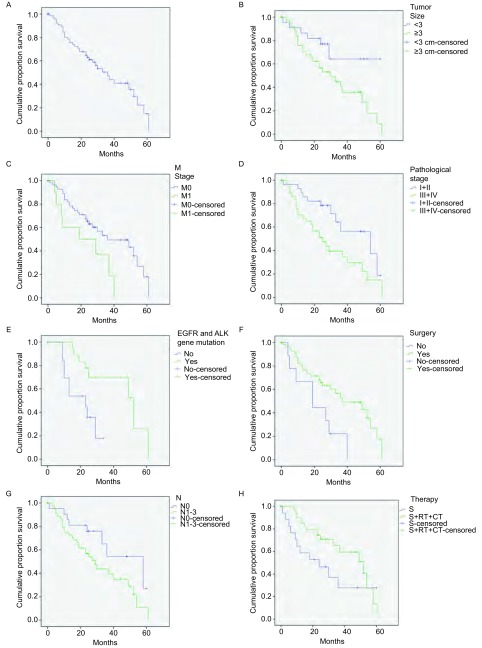
生存曲线图。A：72例肺腺鳞癌患者总生存；B：不同肿瘤长径；C：不同M分期；D：不同病理分期；E：有无*EGFR*和*ALK*基因突变；F：是否手术；G：有无淋巴结转移；H：是否综合治疗。 Survival curves. A: overall survival curves of 72 cases of patients with ASC; B: according to tumor size; C: according to metastasis; D: according to pathological stage; E: Survival of patients with ASC according to *EGFR* and *ALK* gene mutation; F: according to surgery; G: according to lymph nodes metastasis; H: according to multi-modality therapy. S: surgery. RT: radiotherapy. CT: chemotherapy.

### 2.2 复发转移情况

61例接受肺癌手术患者中35例（57.4%）有局部区域复发或远处转移，最短术后2个月疾病复发转移，最长40个月，中位无疾病进展时间为14个月。术后19例发生肺转移，10例纵隔、肺门、锁骨上淋巴结转移。胸外器官远处转移位置以脑部最常见，其次常见骨转移，共9例出现脑转移，6例骨转移，3例肾上腺转移，2例肝转移，1例心包转移，1例腹腔转移。

### 2.3 单因素统计分析肺腺鳞癌的预后因素

本研究对患者性别、年龄、吸烟史、肿瘤长径、侵犯胸膜情况、T分期、N分期、M分期、病理分期（早期和中晚期）、基因突变（*EGFR*和*ALK*突变）、手术、综合治疗等因素对生存的影响进行单因素分析，结果显示肿瘤长径、M分期、肿瘤-淋巴结-转移（tumor-node-metastasis, TNM）病理分期、基因突变、手术对患者预后的影响有统计学意义（表 3，图 1B-图 1F）。肿瘤长径＜3 cm者生存期明显长于肿瘤长径≥3 cm者（*P*=0.038）。无远处转移患者预后优于早期发生远处转移者（*P*=0.038）。早期（Ⅰ期、Ⅱ期）ASC患者生存期明显长于中晚期（Ⅲ期、Ⅳ期）患者（*P*=0.016）。有基因突变组因使用TKI靶向治疗生存率远高于野生型组（*P*=0.007）。手术组患者生存率明显高于未手术组（*P*=0.014）。无区域淋巴结转移组中位生存时间大于有淋巴结转移组（58个月*vs* 29个月，*P*=0.076），但无统计学差异（图 1G）。单纯手术组与手术联合放化疗组虽然统计学上无差异（*P*=0.060），但从生存曲线可以看出综合治疗组患者生存率高于单纯手术组，尚需进一步扩大样本量得出结论（图 1H）。

**3:** 72例肺腺鳞癌的生存单因素分析 Univariate analysis for the survival of 72 cases with ASC

Factor		*n*	3-year survival rate (%)	Median survival time (months)	*P*
Gender	Male	44	44.4	30	0.895
	Female	28	42.9	36	
Age (yr)	< 60	34	52.1	40	0.814
	≥60	38	42.9	30	
Smoke	Yes	39	42.9	30	0.910
	No	33	45.0	37	
Tumor size	< 3 cm	22	64.2	30	0.038
	≥3 cm	44	40.3	36	
Visceral pleura invasion	Yes	51	53.3	37	0.607
	No	7	62.5	52	
Tumor	T1-T2	49	50.9	37	0.118
	T3-T4	23	26.7	23	
Nodes	N0	22	54.2	58	0.076
	N1-N3	50	39.4	29	
Metastasis	M0	61	49.3	36	0.038
	M1	11	18.8	19	
Pathological stage	Ⅰ+Ⅱ	30	56.1	54	0.016
	Ⅲ+Ⅳ	42	34.6	25	
Gene mutation	Yes	19	70.0	52	0.007
	No	14	17.9	23	
Surgery	Yes	61	52.7	37	0.014
	No	11	0	19	
Therapy	Surgery	19	27.8	24	0.060
	Surgery+radiotherapy+chemotherapy	42	59.4	52	

## 3 讨论

ASC是肺癌中一种少见的病理类型，国外报道ASC在原发性肺癌中占0.4%-4.0%^[4]^。本研究显示ASC病例数约占原发性肺癌的0.4%（72/16, 252），与文献报道相符合。

ASC兼具有腺癌和鳞癌二者的临床特征，一方面影像学检查显示肿瘤多数为周围型，符合肺腺癌特点；另一方面，ASC绝大部分患者为重度吸烟人群，肿瘤直径相对较大，与肺鳞癌相似。本组资料中55.6%的患者有吸烟史，且均为重度吸烟者（吸烟指数≥400支/年）；肿瘤长径最小1.5 cm，最大7.9 cm，中位值为3.35 cm；77.8%肺癌原发灶为周围型肿块，这与公认的ASC临床特点一致。

术前明确ASC的病理诊断较困难。本组72例患者中54例行穿刺活检及痰脱落细胞学检查，仅确诊ASC 6例，术前病理确诊率为11.1%（6/54），大部分诊断为单一的组织成分如鳞癌或腺癌。考虑术前病理确诊率低主要与取材量少、穿刺部位局限有关。建议行纤维支气管镜检查或经皮引导下肺穿刺活检并在肿瘤不同部位多点取材，以提高ASC的术前确诊率。

本研究中33例行基因检测，17例（51.5%）*EGFR*突变，2例（2.1%）*KRAS* 12突变，2例（2.1%）*ALK*突变。Tochigi等^[5]^检测23例西方人群ASC患者，*EGFR*突变与*KRAS*突变均为13%（3/23），与西方人群腺癌*EGFR*突变率相近。Sasaki等^[6]^报道日本ASC患者*EGFR*突变率为15%（4/26），韩国ASC患者*EGFR*突变率为44%（11/25）^[7]^。本研究中*EGFR*突变率高于国外文献报道，有待进一步扩大样本量探讨我国ASC基因突变状态。全组患者有13例口服吉非替尼或厄洛替尼或埃克替尼，8例PFS超过6个月，最长20个月，但其中1例因严重皮肤反应停药。提示*EGFR*突变阳性患者可行TKI药物治疗，但需注意药物不良反应。一项54例ALK阳性的非小细胞肺癌和100例*ALK*野生型腺癌的比较研究发现，*ALK*重排更多见于不吸烟的年轻患者，镜下病理常见肿瘤细胞呈粘液性筛状和/或固体印戒细胞表现、缺乏贴壁生长模式，并表达甲状腺转录因子（thyroid transcription factor-1, TTF1）和p63^[8]^。提示可在此类患者中行ALK检测，进而从克唑替尼靶向治疗中获益。本组2例ALK阳性患者年龄分别为43岁和32岁，男女各1例，1例有吸烟史、1例无吸烟史，口服克唑替尼PFS分别为30个月和14个月，疗效明显。因此，肿瘤组织中含有腺癌成分时建议进一步完善*EGFR*、*KRAS*和*ALK*基因检测，从而为辅助治疗提供更多方案。

ASC预后远差于腺癌和鳞癌，特别是在Ⅰ期和Ⅱ期病例中。Bastide等^[9]^在转录子水平进行动物实验显示ASC不是鳞癌和腺癌的简单混合，相对于鳞癌和腺癌，ASC神经内分泌分化和细胞外信号调节激酶增殖途径优先开放，因此有更高的临床侵袭性、易早期转移。研究显示ASC术后患者5年生存率为6.2%-59.4%^[10, 11]^，Mordant等^[3]^报道141例ASC术后患者5年生存率为37%，低于肺鳞癌和腺癌（43.4%和42.8%）。Shimizu等^[12]^分析1, 284例接受手术的原发性肺癌患者，其中ASC 44例，5年生存率为18.5%，明显低于鳞癌和腺癌（38.7%和39.2%）。本组72例患者5年生存率为14.9%，61例手术患者5年生存率为17.8%，与文献报道一致。单因素分析显示肿瘤长径、M分期、TNM病理分期、基因突变、手术对患者预后的影响有统计学意义。无区域淋巴结转移组与淋巴结转移组生存率未显示统计学差异（*P*=0.076），考虑与病例数较少有关，尚需进一步扩大样本量分析。Filosso等^[13]^研究显示辅助治疗是ASC患者预后的独立因素。本研究中综合治疗组（手术联合放化疗）患者生存率高于单纯手术组，但未显示统计学差异（*P*=0.060），与样本量小、随访时间不足够长及失访较多有关。

综上所述，ASC恶性程度高、预后差，对ASC推荐手术为主的综合治疗，个体化分子水平检测指导TKI治疗有助于延长患者生存期。
